# The Effect of Size of Black Cherry Stumps on the Composition of Fungal Communities Colonising Stumps

**DOI:** 10.1515/biol-2019-0054

**Published:** 2019-12-18

**Authors:** Korzeniewicz Robert, Marlena Baranowska, Jolanta Behnke-Borowczyk

**Affiliations:** 1 Poznań University of Life Sciences, Department of Silviculture; ul. Wojska Polskiego 71A, 60-625 Poznań; Poland; 2 Poznań University of Life Sciences, Department of Forest Pathology, ul. Wojska Polskiego 71c, 60-625 Poznań; Poland

**Keywords:** *Prunus serotina*, Illumina System, saprotrophs, invasive species

## Abstract

We investigated fungal communities colonising black cherry stumps. We tested the hypothesis that black cherry stumps of greater diameter should be characterised by more diverse fungal communities than stumps of smaller diameter. The material for analyses came from Podanin Forest District. DNA was extracted using a Plant Genomic DNA purification kit. The results were subjected to bioinformatic analysis and statistical analysis. The OTU sequences were compared using the BLAST algorithm with reference sequences from the UNITE database. In total, 8192 raw sequences were obtained from samples of black cherry stumps applying the Illumina sequencing technique. The results of the statistical analysis indicate a trend towards increased diversity in bigger black cherry stumps. The dominant share of fungi associated with wood decomposition indicates the progressing process of decomposition in stumps. Identification of the role and functions of the individual components of fungal communities colonising stumps may provide insight into the overall ecology of these organisms and provide a basis for improved plant protection, with a view to limiting the occurrence of black cherries in the future in undesirable locations outside their natural range.

## 1 Introduction

Dynamic development of the black cherry (*Prunus serotina*) population has been observed in monocultures of Scots pine (*Pinus sylvestris* L.), plantations of black pine (*P. nigra* Arn.) and European larch (*Larix decidua* Mill.) [1], fresh mixed coniferous forest, fresh mixed forest and fresh forest stands [2, 3]. When appearing on a mass scale in the shrub layer, black cherry hinders regeneration, growth and development of native tree species such as oak or pine, which lose in the competition e.g. for light [1]. For these reasons remedial action is being undertaken to limit the occurrence of black cherry. The methods used to control invasive species are frequently based on experience, rather than on the results of research [4]. Attempts to control black cherry based on methods which are not supported by the results of reliable evidence-based research may be inappropriate, and in the longer term a mistaken strategy, comparable in severity to the original intended introduction of that species [1].

One of the factors leading to the classification of a species as invasive is the lack of organisms that are antagonistic to it in the newly colonised environment [3]. Our current knowledge concerning antagonistic organisms, particularly fungi, in relation to the black cherry is far from satisfactory. In Poland very few studies have been published on the mycological pathogens of this host plant species or more broadly the genus *Prunus* [5, 6, 4]. The most numerous publications concern *Chondrostereum purpureum* (Pers.), which in Western Europe is used in the biological control of undesirable deciduous species, including the black cherry [7, 8, 9]. Observations in the Kampinos National Park provided information on the occurrence of macrofungi on decomposing black cherry wood [10, 4].

However, there are no reports on communities of microfungi colonising black cherry wood. In view of the above it was decided to investigate fungal communities colonising black cherry stumps. Herein, we tested the hypothesis that black cherry stumps of greater diameter should be characterised by more diverse and more numerous fungal communities than stumps of smaller diameter (i). It was also assumed that: the saprotrophs will dominate in the fungal communities of black cherry (ii), the Illumina system will identify the majority of fungi at the level of genus or species (iii), and the month of felling will have an influence on the fungal communities (iv).

## 2 Materials and Methods

The material for analyses consisted of 15 black cherry stumps of maximum 5 cm diameter outside bark (sample K1) and 15 stumps that were over 5 cm in diameter outside bark (sample K2), left after the trees had been felled in March, April and May in the Podanin Forest District (19°28´00˝E 52°04´00˝N, the Margonin Forest Division, compartment 342a) (with 5 stumps in each month). The dominant forest site type was fresh mixed forest (LMśw), growing on a rusty brown soil (RDbr). From the selected stumps 2 cm discs were cut, which were then spot drilled using a SPARKY BUR 15E cordless impact drill with a 2 mm bit. The material collection procedure was performed according to [11]. Samples of pulverised wood were ground in a mortar frozen to –70°C. DNA was extracted using a Plant Genomic DNA purification kit (ThermoScientific). The protocol was modified to include extended lysis. The fungal community was identified to species based on the ITS½ rDNA region. Analysis was conducted using specific primers ITS FI2 5`GAA CCW GCG GAR TCA 3` and 5.8S 5`CGC TGC GTT CTT CAT 3` [12]. The reaction mixture was composed of 2.5 μl DNA, 0.2 μl each primer, 10.6 μl deionised water and 12.5 μl 2X PCR MIX (A&A Biotechnology). The amplification reaction was run in a thermocycler and included initial denaturation (94°C 5 min); 35 cycles of denaturation (94°C 30 s), annealing (56°C 30 s) and elongation (72°C 30 s); and final elongation (72°C 7 min). Next, the product was verified on 1% agarose gel stained with Midori Green Advance DNA (Genetics). The product obtained was purified and sequenced using the SBS technology by Illumina (Genomed S.A. Warszawa).

The results were subjected to bioinformatic analysis (PIPITS, PEAR; FASTX, ITSx, UNITE) and statistical analysis. The OTU sequences were compared using the BLAST algorithm with reference sequences from the UNITE database. Identification was performed to the rank of the lowest possible taxon. A description of the individual stages of the bioinformatic and statistical analyses was given by Szewczyk et al. 2017 [13].

## 3 Results

In total, 8192 raw sequences were obtained from 18 samples of black cherry stumps applying the Illumina sequencing technique. This number includes sequences of culturable fungi (6652 = 81.20%), non-culturable fungi (540 = 6.59%) and organisms with no reference sequence in the database (1001 = 12.21%). The stumps were colonised by 363 taxa. Cultured fungi of small stumps (K1): Ascomycota, Basidiomycota, Glomeromycota and Zygomycota were represented by 1134 (55.06%), 286 (11.8%), 6 (0.25%) and 6 (0.25%) taxa, respectively, comprising 85.15% of all taxa detected. In turn, cultured fungi from big stumps (K2), i.e. Ascomycota, Basidiomycota, Glomeromycota and Zygomycota, were represented by 3245 (56.25%), 1265 (21.93%), 1 (0.02%) and 28 (0.49%) taxa, respectively. Non-culturable organisms were represented by 310 taxa in samples K1 and 335 in samples K2.

**Table:** 

	Spring K1	Spring K2
D-Mg index	13.9807	34.1791
Shannon`s diversity index H	2.4793	3.5573
Shannon`s evenness index E	0.5275	0.6248
Simpson’s diversity index	0.14	0.0731
Berger-Parker Dominance index	0.1258	0.16

Margalef’s index (DMg), Shannon’s diversity index (H’) and Simpson’s diversity index (D) indicate a trend towards increased diversity in bigger black cherry stumps (K2) (Table 1). Similarly, the dominance of single taxa in communities in larger stumps (K2) resulted in low values for Shannon’s evenness index (E) and high values for Berger–Parker’s dominance index (d).

The most common fungi in small stumps (K1) included *Pleurophoma ossicola* (25.46%), *Mycena megaspora* (5.49%), *Trichosporon otae* (3.26%), *Penicillium citreonigrum* (2.93%), *Yarrowia lipolytica* (2.06%), *P. lapidosum* (2.35%) *Blastobotrys* sp. (2.02%), and *Candida fructus* (1.98%). However, in larger stumps (K2) the most common fungi were *Proliferodiscus* sp. (14.75%), *Laetiporus sulphureus* (3.73%), *Tumularia* sp. (2.24%), *Cuniculitrema polymorpha* (1.84%), *Curvibasidium cygneicollum* (1.61%), *C. mycetangii* (1.42%), *Biatora sphaeroidizax* (1.37%), *Rhizoscyphus sp*. (1.32%), *Fellozyma inositophila* (1.23%), *Hamamotoa lignophila* (1.04%) (Tab. 2).

The fungi found on both small and large stumps were *Beauveria pseudobassiana, Chalara* sp., *Ciborinia candolleana, Dictyochaeta* sp., *Infundichalara minuta, Jattaea ribicola, Lachnellula calyciformis, Penicillium bialowiezense, P. citreonigrum, P. lapidosum, P. raphiae, Phialocephala compacta, Pleurophoma ossicola, Proliferodiscus* sp., *Sordariomycetes* sp., *Tumularia* sp., *Agaricomycetes* sp., *Microstroma album, Mycena megaspora, Vishniacozyma victoriae, Rozellomycota* sp. and *Umbelopsis isabellina*.

## 4 Discussion

Greater diversity of fungal species in the community was observed for black cherry stumps exceeding 5 cm in diameter. In both cases the fungal community was dominated by fungi from the Phylum Ascomycota, with their share slightly exceeding 55% in the analysed communities, as confirmed by earlier reports concerning deciduous trees [14, 15]. These results indicate that the dominance of Ascomycota in the fungal community associated with dead wood is also related to the degree of its decomposition, i.e. the earlier the decomposition stage of wood, the greater the share of Ascomycota in the community [16, 17, 18, 19, 20]. The analysed stumps were classified into wood decomposition class 1 and samples were collected 1 year after the black cherries were removed from the stand, thus the recorded results confirm earlier reports. Fungi belonging to the Phylum Ascomycota cause slow wood decomposition, which is limited only to surface decay in periods of increased humidity. However, alternating drought and wet periods promote deeper penetration of the mycelium and lead to extended wood decomposition [21]. In turn, in the analysed community the share of taxa belonging to the Phylum Basidiomycota was almost 2-fold greater in the community of black cherry stumps with diameters exceeding 5 cm than in black cherry stumps with diameters not exceeding 5 cm. A lesser share was recorded for taxa belonging to the Phylum Basidiomycota. Similar results were also reported by van der Wall et al. 2015 [22] and Kwaśna et al. 2016 [15].

*Pleurophoma ossicola* was the taxon found most frequently on black cherry stumps of lesser diameter (over 25%), although it was also recorded to some extent on larger stumps (0.23%). It was found in a stand with Scots pine in Germany [23]. The literature lacks data on the function of this fungus in the community. The rotting bonnet fungus (*Mycena megaspora*) was one of the most abundant species recorded in the fungal community of black cherry stumps (K1, 5.49%), as well as a species common for both analysed variants (K1 and K2). Fungi belonging to that genus are most frequently classified as saprotrophs, except for *M. citricolor* (Ber. & Curt.). Fungi from the genus *Mycena* are commonly found on dead wood of coniferous trees and angiosperms, on decomposing stems and branches, on the bark of living trees, in soil, and less frequently on decomposing ferns, grasses or other herbaceous plants and mosses [24].

In the fungal community of black cherry stumps of over 5 cm in diameter (K2) the most abundant taxon was *Proliferodiscus*, which was a common taxon for both analysed black cherry communities. Fungi from that genus play an important role in the decomposition of various organic substances, including dead wood, branches and leaf litter. An example is provided by *P. pulveraceus*, a new species in Poland discovered in 2008, which is found on dead hornbeam wood [25].

*Beauveria pseudobassiana* was a common species in both analysed communities; nevertheless, its share was below 1%. This genus includes *B. bassiana* and *B. brongniartii*, used in biological control of harmful insects [26]. The genus *Chalara* was also found to be a common taxon for both communities, comprising pathogens such as *Ch. fraxinea* causing ash die-back [27,28]. Other taxa recorded in both communities were *Ciborinia candolleana*, *Dictyochaeta*, and *Infundichalara minuta*, which is classified as a saprotrophic species [29, 30, 31]. *Lachnellula calyciformis* was another species common in both communities; as a saprotroph it colonises knots, snags, dead branches and twigs, and, less commonly, living trees [32]. Other species common for both communities of black cherry stumps include *Penicillium bialowiezense*, which so far has been isolated from forest soil (in Poland), as well as *P. raphiae* found in soil [33]. In both cases *Microstroma album* was identified, which is classified as an obligate parasite of *Quercus* [34].

The available literature still lacks reports thoroughly detailing communities of fungi colonising black cherry stumps. Information on fungi on roots of that species and studies of Macromycetes colonising black cherry wood have been published by Kwaśna et al. 2008 [35]. Similarly, as reported by Kwaśna et al. 2008 [35], in the current study of the community of fungi colonising black cherry stumps species from the genus *Mycena* were recorded, e.g. *M. cinerella, M. galericulata, M. megaspora* and *M. sanguinolenta*. In the fungal community colonising stumps exceeding 5 cm, similarly to the study by Kwaśna et al. 2008 [35], we found a small group of fungi from the genus *Fusarium* and a single species *F. cyanostomum*, as well as *Humicola* spp. *Sporothrix dimorphospora*. In stumps of less than 5 cm in diameter a fungal species from the genus *Trichoderma* was identified: *T. asperellum*. In wood of stumps of all black cherry trees, fungi from the genus *Penicillium* were identified, although this community differed from that reported in black cherry roots. In black cherry stumps the following *Penicillium* fungi were found*: P. angulare, P. bialowiezense, P. citreonigrum, P. kongii, P. lanosum, P. lapidosum, P. miczynskii, P. raphiae* and *P. viticola*. Identification of fungal communities in black cherry roots and stumps was not consistent due to the differences in the analysed material and the methods applied to identify the respective communities. In the Kampinos National Park in the wood of black cherries subjected to mechanical control, analysis showed the presence of *Nectria cinnabarina* (Tode) Fr. anamorph [4], while in the case analyses of stumps a sparse share (>1%) of Nectriaceae was found. Other differences were found in the species *Mycena galericulata* [4], which was also identified on stumps with diameters of less than 5 cm, and *M. haematopus* (Pers.) P. Kumm; *Peniophora cinerea* (Pers.) Cooke; *Phaeotremella pseudofoliacea* Rea and *Stereum rugosum* [4], which we identified in the wood of larger stumps. *Stereum rugosum* was only recorded in approximately 2% of trees, but accounted for approximately 7% of trees which were colonised by fungi. This species is mainly saprotrophic in character. Locally it causes bark necroses or cankers on stems of deciduous trees [36]. In the Kampinos National Park *Laetiporus sulphureus* has been reported on logs, branches and trees of the black cherry [4], while in this study it had a 3.76% share in wood of stumps with diameters larger than 5 cm. *Stereum hirsutum* was identified in this study in the wood of larger black cherry stumps, as well as *Tremella mesenterica* Retz [4], whereas in our study a share of the genus *Tremella* was identified in this community.

## 5 Conclusion

The results of the above-mentioned study are consistent with our hypothesis that larger black cherry stumps should be characterised by a more diverse fungal species composition both qualitatively and quantitatively. Taking into account this study’s results, it seems justified to undertake further studies on the species *Pleurophoma ossicola*, whose share in black cherry stumps with diameters of maximum 5 cm exceeded 25%, while its ecology and function in the forest environment have not been thoroughly identified to date.

Saprotrophs and pathogens, both termed facultative parasites, that are primarily found in the analysed black cherry stumps include *Proliferodiscus* sp., *Laetiporus sulphureus, Mycena megaspora, Trichosporon otae*, *Yarrowia lipolytica, Tumularia* and *Curvibasidium cygneicollum*. The dominant share of fungi associated with wood decomposition indicates the progressing process of decomposition in stumps; however, the rate of black cherry wood decomposition by the above-mentioned taxa has not been determined. In the fungal community of black cherry stumps we did not find any economically important pathogens associated with tree root systems, for example genera such as *Armillaria* and *Heterobasidion*. Using the criterion of a 1% share in the community, we recorded the presence of a mycorrhizal fungus *Rhizoscyphus* sp. associated with the family Ericaceae. Moreover, we also identified fungi which to date have been considered to have no economic importance in the forest economy.

The applied sequencing method based on the Illumina System made it possible to identify most fungi (nearly 90%) to the genus or species levels. Classification of fungi was more effective than in studies based on 454 sequencing, in which 40% sequences were unidentified even at the genus level [19,20]. This confirms the efficacy of the applied method for determining and defining the composition of fungal communities.

The analysis of the quantitative and qualitative composition undertaken in our study on fungal communities colonising black cherry stumps is in line with basic research on this species. Identification of the role and functions of the individual components of fungal communities colonising stumps may provide some insight into the overall ecology of these organisms and provide a basis for improved plant protection and control, with a view to limiting the occurrence of black cherries in the future in undesirable locations outside their natural range. Our study is an introduction into an analysis of variability in the structure of the above-mentioned community.

## Appendix

**Table:**

No.	Taxon	K1	K2
		
		%	%
**Fungi**			
**Ascomycota**			
1.	*Absconditella* sp.	0.000	0.017
2.	*Acephala applanata* Grünig & T.N. Sieber	0.124	0.000
3.	*Alatospora* sp.	0.000	0.017
4.	*Arachnopeziza* sp.	0.000	0.312
5.	*Articulospora* sp.	0.000	0.017
6.	Ascomycota	12.629	15.999
7.	*Barssia maroccana* G. Moreno, Manjón, Carlavilla & P. Alvarado	0.124	0.000
8.	*Beauveria pseudobassiana* S.A. Rehner & Humber	0.083	0.104
9.	*Biatora sphaeroidiza* Printzen & Holien	0.000	1.369
10.	Bionectriaceae	0.000	0.017
11.	*Blastobotrys* sp.	2.022	0.000
12.	*Cadophora luteo-olivacea* (J.F.H. Beyma) T.C. Harr. & McNew	0.000	0.087
13.	*Caliciopsis beckhausii* (Körb.) Garrido-Ben. & Pérez-Ort.	0.000	0.052
14.	*Candida fructus* (Nakase) S.A. Mey. & Yarrow +*C. mycetangii* Kurtzman+ *Candida* sp.	1.981	1.487
15.	*Capronia pilosella* (P. Karst.) E. Müll., Petrini, P.J. Fisher, Samuels & Rossman + *C. pulcherrima* (Munk) E. Müll., Petrini, P.J. Fisher, Samuels & Rossman+ *Capronia* sp.	0.000	0.087
16.	*Cephalosporium* sp.	0.000	0.017
17.	Cephalothecaceae	0.083	0.503
18.	*Chaetomium* sp.	0.000	0.017
19.	Chaetothyriales	0.000	0.589
20.	*Chalara* sp.	0.041	0.017
21.	*Chloridium* sp.	0.124	0.000
22.	*Ciborinia candolleana* (Lév.) Whetzel	0.041	0.017
23.	*Ciliophora* sp.	0.124	0.000
24.	*Cladophialophora arxii* Tintelnot + *Cladophialophora* sp.	0.000	0.364
25.	Claussenomyces	0.000	0.017
26.	*Collophora* sp.	0.000	0.104
27.	*Colpoma quercinum* (Pers.) Wallr.	0.000	0.017
28.	*Coniochaeta* sp.	0.000	0.121
29.	*Crocicreas epicalamia* (Fuckel) Raitv. & Kutorga + *Crocicreas* sp.	0.206	0.017
30.	*Cyphellophora reptans* (de Hoog) Réblová & Unter.	0.000	0.191
31.	Dermateaceae	0.206	0.052
32.	*Desertella* sp.	0.000	0.156
33.	*Diaporthe helicis* Niessl	0.000	0.035
34.	*Dictyochaeta* sp.	0.165	0.017
35.	*Discosia pseudoartocreas* Crous & Damm	0.000	0.069
36.	*Discostroma* sp.	0.000	0.069
37.	*Exophiala bergeri* Haase & de Hoog + *E. castellanii* Iwatsu, Nishim. & Miyaji + *E. psychrophila* O.A. Pedersen & Langvad +*E. sideris* Seyedm. & de Hoog +*Exophiala* sp.	0.000	1.005
38.	*Fusarium cyanostomum* (Sacc. & Flageolet) O’Donnell & Geiser +*Fusarium* sp.	0.000	1.004
39.	*Fusicladium cordae* Koukol	0.000	0.017
40.	*Geomyces auratus* Traaen	0.000	0.035
41.	Helotiaceae	0.000	0.624
42.	Helotiales	1.197	0.312
43.	Herpotrichiellaceae sp.	0.041	2.704
44.	*Humicola* sp.	0.000	0.416
45.	*Hyalorbilia inflatula* (P. Karst.) Baral & G. Marson	0.000	0.017
46.	Hyaloscyphaceae	0.000	0.035
47.	*Hydnotrya tulasnei* (Berk.) Berk. & Broome	0.041	0.000
48.	*Hyphodiscus hymeniophilus* (P. Karst.) Baral	0.000	0.052
49.	Hypocreales	0.165	0.537
50.	*Hypomyces lactifluorum* (Schwein.) Tul. & C. Tul.	0.000	0.017
51.	*Infundichalara minuta* Koukol	0.206	0.052
52.	*Jattaea aphanospora* Réblová & J. Fourn.	0.000	0.104
53.	*Jattaea ribicola* Réblová & Jaklitsch	0.041	0.035
54.	*Junewangia queenslandica* (Matsush.) J.W. Xia & X.G. Zhang	0.000	0.069
55.	*Lachnellula calyciformis* (Batsch) Dharne	0.413	0.156
56.	*Lecania* sp.	0.000	0.017
57.	*Lecanicillium muscarium* (Petch) Zare & W. Gams	0.000	0.035
58.	Lecanorales	0.000	0.017
59.	Lecanoromycetes	0.000	0.329
60.	*Lecophagus* sp.	0.000	0.347
61.	Leotiomycetes	0.083	0.988
62.	*Lepraria elobata* Tønsberg	0.000	0.069
63.	*Leptodontidium trabinellum* (P. Karst.) Baral, Platas & R. Galán	0.000	0.329
64.	*Lophium arboricola* (Buczacki) Madrid & Gené	0.000	0.052
65.	*Lophodermium pinastri* (Schrad.) Chevall.	0.454	0.000
66.	*Menispora manitobaensis* B. Sutton	0.000	0.156
67.	*Metapochonia bulbillosa* (W. Gams & Malla) Kepler, S.A. Rehner & Humber	0.000	0.087
68.	*Micarea assimilata* (Nyl.) Coppins	0.000	0.017
69.	*Mollisia cinerea* (Batsch) P. Karst.	0.000	0.017
70.	*Mycoleptodiscus* sp.	0.000	0.035
71.	Nectriaceae	0.000	0.052
72.	*Neofabraea* sp.	0.000	0.087
73.	*Oidiodendron majus* G.L. Barron	0.000	0.017
74.	Onygenaceae	0.000	0.052
75.	*Ophiostoma tsotsi* Grobbel., Z.W. De Beer & M.J. Wingf.si	0.000	0.069
76.	Ophiostomataceae	0.000	0.087
77.	*Orbilia aprilis* Velen. + *O.aristata* (Velen.) Velen.	0.000	0.156
78.	*Orbiliomycetes* sp.	0.000	0.052
79.	*Otidea subterranea* Healy & M.E. Sm.	0.000	0.069
80.	*Pannaria athroophylla* (Stirt.) Elvebakk & D.J. Galloway	0.000	0.087
81.	*Parmelia subdivaricata* Asahina	0.000	0.017
82.	*Penicillium angulare S.W. Peterson, E.M. Bayer & Wicklow + P. bialowiezense K.W. Zaleski + P. citreonigrum Dierckx + P. kongii L. Wang + P. lanosum Westling + P. lapidosum Raper & Fennell + P. miczynskii K.W. Zaleski P. raphiae Houbraken, Frisvad & Samson + P. viticola Nonaka & Masuma*	5.365	1.144
83.	*Pezicula sporulosa* Verkley	0.000	0.191
84.	*Phacidium grevilleae* Crous & M.J. Wingf.	0.000	0.173
85.	*Phaeomollisia piceae* T.N. Sieber & Grünig	0.000	0.035
86.	*Phaeomoniella* sp.	0.000	0.017
87.	*P. compacta* Kowalski & Kehr + *P. glacialis* Grünig & T.N. Sieber + *P. scopiformis* Kowalski & Kehr + *Phialocephala* sp.	0.330	0.572
88.	*Picoa juniperi* Vittad.	0.000	0.676
89.	*Pleurophoma ossicola* Crous, Krawczynski & H.-G. Wagner	25.464	0.225
90.	*Proliferodiscus* sp.	0.413	14.751
91.	Pseudeurotiaceae	0.000	0.035
92.	*Pseudogymnoascus verrucosus* A.V. Rice & Currah	0.000	0.451
93.	*Rhizoscyphus* sp.	0.000	1.317
94.	Saccharomycetales	0.000	0.260
95.	*Sarea resinae* (Fr.) Kuntze	0.000	0.069
96.	*Sarocladium strictum* (W. Gams) Summerb.	0.000	0.711
97.	Sordariales	0.000	0.017
98.	*Sordariomycetes* sp.	0.083	0.416
99.	*Sporothrix dimorphospora* (Roxon & S.C. Jong) Madrid, Gené, Cano & Guarro	0.000	0.208
100.	*Stachybotrys* sp.	0.000	0.260
101.	*Talaromyces amestolkiae* N. Yilmaz, Houbraken, Frisvad & Samson + *T. verruculosus* (Peyronel) Samson, N. Yilmaz, Frisvad & Seifert *+ T. wortmannii* C.R. Benj.	0.165	0.070
102.	*Taphrina confusa* (G.F. Atk.) Giesenh.	0.000	0.035
103.	*Tolypocladium* sp.	0.000	0.087
104.	*Trichoderma asperellum* Samuels, Lieckf. & Nirenberg	0.371	0.000
105.	*Tridentaria implicans* Drechsler	0.000	0.035
106.	*Trimmatostroma cordae* N.D. Sharma & S.R. Singh	0.000	0.017
107.	*Truncatella restionacearum* S.J. Lee & Crous	0.000	0.087
108.	*Tumularia* sp.	0.083	2.236
109.	Valsaceae	0.041	0.347
110.	*Venturia hystrioides* (Dugan, R.G. Roberts & Hanlin) Crous & U. Braun +Venturia sp.	0.000	0.168
111.	Venturiaceae	0.000	0.035
112.	Venturiales	0.000	0.069
113.	*Xenopolyscytalum pinea* Crous	0.000	0.017
114.	Xylariaceae	0.083	0.000
115.	*Yamadazyma mexicana* (M. Miranda, Holzschu, Phaff & Starmer) Billon-Grand	0.000	0.052
116.	*Yarrowia lipolytica* (Wick., Kurtzman & Herman) Van der Walt & Arx	2.064	0.000
	**Frequency of Ascomycota**	**55.056**	**56.249**
**Basidiomycota**			
1.	Agaricaceae	0.083	0.035
2.	Agaricales	0.165	0.052
3.	*Agaricomycetes* sp.	0.041	0.017
4.	Agaricostilbales	0.000	0.017
5.	*Amanita parcivolvata* (Peck) E.-J. Gilbert	0.000	0.017
6.	Auriculariales	0.041	0.000
7.	Basidiomycota	0.537	1.161
8.	*Bullera* sp.	0.000	0.017
9.	*Bulleromyces albus* Boekhout & Á. Fonseca	0.000	0.017
10.	Cantharellales	0.083	0.000
11.	*Chionosphaera cuniculicola* R. Kirschner, Begerow & Oberw.	0.000	0.017
12.	Chrysozymaceae	0.000	0.017
13.	*Clitopilus hobsonii* (Berk.) P.D. Orton	0.000	0.052
14.	*Colacogloea philyla* (Van der Walt, Klift & D.B. Scott) Q.M. Wang, F.Y. Bai, M. Groenew. & Boekhout	0.000	0.087
15.	Colacogloea	0.000	0.052
16.	*Corticium confine* Bourdot & Galzin	0.000	0.017
17.	*Cryptococcus pseudolongus* M. Takash., Sugita, Shinoda & Nakase + C. psychrotolerans V. de García, Zalar, Brizzio, Gunde-Cim. & Van Broock + Cryptococcus sp.	0.041	0.671
18.	*Cuniculitrema polymorpha* R. Kirschner & J.P. Samp.	0.000	1.837
19.	*Curvibasidium cygneicollum* J.P. Samp.	0.000	1.612
20.	Cystobasidiomycetes	0.000	0.069
21.	*Cystobasidium pinicola* (F.Y. Bai, L.D. Guo & J.H. Zhao) Yurkov, Kachalkin, H.M. Daniel, M. Groenew., Libkind, V. de Garcia, Zalar, Gouliamova, Boekhout & Begerow	0.000	0.537
22.	Cystofilobasidiales	0.000	0.173
23.	*Cystofilobasidium infirmominiatum* (Fell, I.L. Hunter & Tallman) Hamam., Sugiy. & Komag. +C. macerans J.P. Samp.	0.000	0.069
24.	*Dacrymyces chrysospermus* Berk. & M.A. Curtis	0.000	0.485
25.	*Dioszegia fristingensis* Á. Fonseca, J. Inácio & J.P. Samp.	0.000	0.017
26.	Erythrobasidiales	0.000	0.069
27.	*Erythrobasidium* sp.	0.000	0.052
28.	*Exobasidium arescens* Nannf. + *E. maculosum* M.T. Brewer + *Exobasidium* sp.	0.000	0.624
29.	*Fellomyces horovitziae* Spaaij, G. Weber & Oberw. + *F. mexicanus* Lopandić, O. Molnár & Prillinger + *Fellomyces* sp.	0.000	0.069
30.	*Fellozyma inositophil*a (Nakase & M. Suzuki) Q.M. Wang, F.Y. Bai, M. Groenew. & Boekhout	0.000	1.231
31.	*Fibulobasidium murrhardtense* J.P. Samp., Gadanho, M. Weiss & R. Bauer	0.000	0.035
32.	*Filobasidium stepposum* (Golubev & J.P. Samp.) Xin Zhan Liu, F.Y. Bai, M. Groenew. & Boekhout	0.000	0.087
33.	*Genolevuria amylolytica* (Á. Fonseca, J. Inácio & Spenc.-Mart.) Xin Zhan Liu, F.Y. Bai, M. Groenew. & Boekhout	0.000	0.017
34.	*Hamamotoa lignophila* (I. Dill, C. Ramírez & A.E. González) Q.M. Wang, F.Y. Bai, M. Groenew. & Boekhout	0.000	1.040
35.	Hydnaceae	0.083	0.000
36.	Hygrophoraceae	0.083	0.017
37.	Hymenochaetales	0.124	0.000
38.	*Inocybe* sp.	0.000	0.884
39.	*Itersonilia pannonica* (Niwata, Tornai-Leh., T. Deák & Nakase) Xin Zhan Liu, F.Y. Bai, J.Z. Groenew. & Boekhout	0.000	0.156
40.	*Kockovaella machilophila* Cañ.-Gib., M. Takash., Sugita & Nakase	0.000	0.676
41.	*Kondoa aeria* Á. Fonseca, J.P. Samp. & Fell	0.000	0.017
42.	*Kriegeria eriophori* Bres. 1891	0.000	0.156
43.	Kurtzmanomyces	0.000	0.035
44.	*Kwoniella pini* (Golubev & I. Pfeiff.) Xin Zhan Liu, F.Y. Bai, M. Groenew. & Boekhout	0.000	0.139
45.	*Laetiporus sulphureus* (Bull.) Murrill	0.000	3.727
46.	Leucosporidiales	0.000	0.017
47.	*Leucosporidiella creatinivora* (Golubev) J.P. Samp.	0.000	0.139
48.	*Leucosporidium drummii* Yurkov, A.M. Schäfer & Begerow + *L. fasciculatum* Babeva & Lisichk. + *Leucosporidium* sp.	0.000	0.416
49.	*Luellia recondita* (H.S. Jacks.) K.H. Larss. & Hjortstam	0.000	0.121
50.	*Malassezia restricta* E. Guého, J. Guillot & Midgley	0.371	0.000
51.	*Mastigobasidium intermedium* Golubev	0.000	0.017
52.	Microbotryomycetes	0.000	0.485
53.	*Microsporomyces pini* (C.H. Pohl, M.S. Smit & Albertyn) Q.M. Wang, F.Y. Bai, M. Groenew. & Boekhout	0.000	0.104
54.	*Microstroma album* (Desm.) Sacc.	0.330	0.087
55.	*Mrakia frigida* (Fell, Statzell, I.L. Hunter & Phaff) Y. Yamada & Komag.	0.000	0.017
56.	*Mycena cinerella* (P. Karst.) P. Karst. + *M. galericulata* (Scop.) Gray + *M. megaspora* Kauffman + *M. sanguinolenta* (Alb. & Schwein.) P. Kumm.	6.108	0.156
57.	*Oberwinklerozyma yarrowii* (Á. Fonseca & Uden) Q.M. Wang, F.Y. Bai, M. Groenew. & Boekhout	0.000	0.017
58.	*Papiliotrema perniciosa* (Golubev, Gadanho, J.P. Samp. & N.W. Golubev) Xin Zhan Liu, F.Y. Bai, M. Groenew. & Boekhout	0.000	0.121
59.	*Peniophora pini* (Schleich. ex DC.) Boidin	0.000	0.017
60.	*Phaeotremella skinneri* (Phaff & Carmo Souza) Yurkov & Boekhout,	0.000	0.017
61.	*Rhodotorula glutinis* (Fresen.) F.C. Harrison + *R. nothofagi* C. Ramírez & A.E. González + *Rhodotorula* sp.	0.000	0.329
62.	Russulales	0.041	0.017
63.	*Schizophyllum* sp.	0.000	0.017
64.	*Septobasidium broussonetiae* C.X. Lu, L. Guo & J.G. Wei + *S. pallidum* Couch ex L.D. Gómez & Henk	0.000	0.130
65.	*Slooffia tsugae* (Phaff & Carmo Souza) Q.M. Wang, F.Y. Bai, M. Groenew. & Boekhout	0.000	0.035
66.	Sporidiobolales	0.000	0.156
67.	*Stereum hirsutum* (Willd.) Pers. + *S. rugosum* Pers.	0.000	0.034
68.	*Tausonia pullulans* (Lindner) Xin Zhan Liu, F.Y. Bai, J.Z. Groenew. & Boekhout	0.000	0.624
69.	Thelephorales	0.000	0.035
70.	*Tremella globispora* D.A. Reid + *T. indecorata* Sommerf. + *Tremella* sp.	0.000	0.416
71.	Tremellales	0.000	0.659
72.	Tremellomycetes	0.000	1.179
73.	*Trichosporon otae* Sugita, Takshima & Kikuchi	3.260	0.000
74.	*Tulasnella* sp.	0.330	0.000
75.	*Vishniacozyma carnescens* (Verona & Luchetti) Xin Zhan Liu, F.Y. Bai, M. Groenew. & Boekhout + *V. victoriae* (M.J. Montes, Belloch, Galiana, M.D. García, C. Andrés, S. Ferrer, Torr.-Rodr. & J. Guinea) Xin Zhan Liu, F.Y. Bai, M. Groenew. & Boekhout	0.083	0.208
76.	*Vonarxula javanica* (Arx & Weijman) Q.M. Wang, F.Y. Bai, M. Groenew. & Boekhout	0.000	0.087
77.	*Yunzhangia auriculariae* (Nakase) Q.M. Wang, F.Y. Bai, M. Groenew. & Boekhout	0.000	0.017
	**Frequency of Basidiomycota**	**11.804**	**21.928**
**Zygomycota**			
1.	*Mortierella hyalina* (Harz) W. Gams	0,247627	
2.	*Umbelopsis isabellin*a (Oudem.) W. Gams	0,247627	
**Others**			
**Plantae**			
1.	Anthophyta	0	0,034668
2.	Chlorophyta	0,288898	1,716069
3.	Plantae	0,123813	0,225342
**Protista**			
1.	Cercozoa sp.	0,165085	0,554689
**Frequency of Oters**			
1.	**No sequence in the database UNITE**	19,27363	12,98319
2.	**Non-cultivable fungi**	12,79406	5,806899
3.	**Number of isolates**	**100**	**100**
4.	**Number of fungi isolates**	**80,14858**	**84,48605**
